# A voice and speech corpus of patients who underwent upper airway surgery in pre- and post-operative states

**DOI:** 10.1038/s41597-024-03540-5

**Published:** 2024-07-09

**Authors:** Estefanía Hernández-García, Alejandro Guerrero-López, Julián D. Arias-Londoño, Juan I. Godino-Llorente

**Affiliations:** 1 Department of Otorhinolaryngology, Hospital Universitario de Fuenlabrada, Fuenlabrada, Madrid Spain; 2 Escuela Técnica Superior de Ingenieros de Telecomunicación, Universidad Politécnica de Madrid, 28040 Madrid, Spain

**Keywords:** Oral manifestations, Biomedical engineering

## Abstract

Many research articles have explored the impact of surgical interventions on voice and speech evaluations, but advances are limited by the lack of publicly accessible datasets. To address this, a comprehensive corpus of 107 Spanish Castilian speakers was recorded, including control speakers and patients who underwent upper airway surgeries such as Tonsillectomy, Functional Endoscopic Sinus Surgery, and Septoplasty. The dataset contains 3,800 audio files, averaging 35.51 ± 5.91 recordings per patient. This resource enables systematic investigation of the effects of upper respiratory tract surgery on voice and speech. Previous studies using this corpus have shown no relevant changes in key acoustic parameters for sustained vowel phonation, consistent with initial hypotheses. However, the analysis of speech recordings, particularly nasalised segments, remains open for further research. Additionally, this dataset facilitates the study of the impact of upper airway surgery on speaker recognition and identification methods, and testing of anti-spoofing methodologies for improved robustness.

## Background & Summary

The vocal tract involves structures from the vocal folds to the lips and from the nasal cavity to the nostrils. Changes in any of these structures may theoretically affect the acoustic properties of the sound emitted^1,2^. Various common pathologies can alter the vocal tract. For example, nasal obstruction can result from various pathologies, such as septal deviation or nasal polyposis^3–5^. The shape, curvature, and relationship of the septal cartilage to the vomer bone affect the airflow through the nostrils and the reverse airflow from the velum to the nasal fossa. This deformity causes airflow resistance, leading to breathing difficulties and altered nasal resonance during speech sound production^6^. The most common surgical treatment for septal deviation is septoplasty, which involves intranasal correction of deformities. This procedure can alter the anatomy of the vocal tract, affecting both objective and subjective voice properties, including nasality, as demonstrated by various authors^6–14^. Regarding nasal polyposis, functional endoscopic sinus surgery (FESS) is the most common procedure. It involves the removal of the inflammatory nasal mucosa and ethmoid bone cells, significantly improving nasal airflow and reducing rinolalia. Furthermore, these changes in the nasal opening and sinus cavity can affect the acoustic resonance, potentially altering the patient’s self-perception of their speech as previously studied^5,7,15–18^. Another example may be recurrent tonsillitis, which is often treated with tonsillectomy, a surgical procedure that involves the removal of the tonsils. This surgery has the potential to affect the anatomy of the vocal tract, resulting in alterations in both objective and subjective voice characteristics, including resonance, as observed in numerous studies^19–24^.

Diverse research papers have explored the pathologies that affect the vocal tract and the effects of surgical interventions on subjective and objective characteristics of the voice and speech, as mentioned previously. However, a notable limitation in this area is the lack of publicly available corpora, which hinders the reproducibility of the previous results. In particular, of the 22 articles previously cited that examined the impact of upper respiratory tract surgery, none of them made their datasets publicly accessible, which poses a significant challenge in replicating their findings. And, to the best of the author’s knowledge, there are no open corpora with these characteristics.

To solve this problem, an extensive database featuring 107 Spanish Castillian speakers was recorded. The corpus contains the voice and speech of the control speakers and patients who underwent a surgical procedure of the upper airway in the pre- and post-operative stages. The surgeries considered are the following: Tonsillectomy, FESS, and Septoplasty. These surgeries were performed by a single surgeon. The dataset comprises a variety of audio materials, including: (i) approximately 2-seconds of sustained vowels for /a/, /e/, /i/, /o/ and /u/, (ii) three additional utterances of approximately 2-seconds for the sustained vowel /a/, (iii) four Text-Dependent Utterances (TDU) each lasting approximately 10 seconds, and (iv) free speech monologues with durations of around 1 minute which were also manually transcribed. In addition to the raw audio files in WAV format, the corpus contains a set of precomputed voice quality measurements featuring the voice recordings. In addition, supplementary metadata was associated with voice and speech recordings, such as clinical data, demographic data, and doctor’s comments on both patients and audios.

Audio recordings were conducted over three sessions: (a) approximately two weeks before surgery (on average, 8.32 days prior), (b) approximately two weeks after surgery (on average, 14.73 days post-operation), and (c) three months following the respective surgical procedures (on average, 78.5 days after). The control group underwent recordings within the same time intervals. After each hospital visit, thorough clinical data annotation covered: (i) patient weight; (ii) nasality questionnaire results; (iii) nasometry measurements; (iv) perceptual scores using GRBAS (Grade, Roughness, Breathiness, Asthenia, Strain)^25^; and (v) detailed comments and observations recorded by the attending physician during the visit.

Furthermore, demographic data was recorded in the first session, including: (i) sex; (ii) stature; (iii) diagnosis; (iv) smoking status; (v) presence of Obstructive Sleep Apnea (OSA); (vi) use of Continuous Positive Airway Pressure (CPAP) devices; (vii) occupation, involving voice use; (viii) tonsillar grade for tonsillectomy patients; (ix) surgery date; (x) doctor’s annotations; and (xi) Computer Tomography (CT) Sinuses Lund-Mackay score^26^ (when applicable).

Several subsets of this corpus have undergone prior analysis. Hernández *et al*.^27^ examined the Septoplasty group, revealing significant differences after septoplasty in nasalance, GRBAS, and nasality questionnaire compared to the control group across vowels. The FESS cohort, studied by Hernández *et al*.^28^, exhibited significant post-FESS changes in GRBAS and a slight decrease in fundamental frequency (F0). Furthermore, the same study presents results using an automatic speaker identification system, reporting a higher equal error rate for the FESS group compared to the control group, indicating FESS-induced vocal tract alterations that led to increased speaker recognition errors. Lastly, Moro *et al*.^24^ analysed the robustness of different speaker recognition schemes with respect to preoperative and postoperative changes, underscoring the importance of updating speaker enrolment data after surgery for optimal performance of automatic speaker recognition systems.

This extensive data set, which includes a total of 3,800 audio files with an average of 35.51 ± 5.91 audio recordings per patient, provides the scientific community with the tools to conduct a systematic investigation of the objective repercussions of upper respiratory tract surgery on the voice, across various surgical procedures, and also to study speaker verification/identification spoofing countermeasures.

## Methods

The corpus contains audio recordings in WAV format for each patient: (a) two weeks before surgery, (b) two weeks after, and (c) three months after the aforementioned surgical procedures. The same procedure was followed for the control group, but in this case, the surgery was unrelated to the vocal tract. The speech is accompanied by the estimation of the three first formant contours as sequences (in Python®-compatible PKL format) and their plot over the spectrograms (in PNG format). An acoustic analysis^29^ was also performed, including the most significant voice quality measurements typically used in the literature about voice quality (in CSV format).

This section is structured as follows. First, an overview of the demographics of the participants and the inclusion criteria is provided. The clinical data are then presented, providing insight into the systematic collection process during Session (Ses.) 1. Following this, the Audio Registration section delves into the audio registration process, detailing the creation of the script for the audios, recording session specifics, and the extraction of audio features. Lastly, ethical considerations and declarations are thoroughly addressed in the Ethical Declaration section, ensuring transparency and adherence to ethical guidelines throughout the study.

### Participants

Speech data from 107 Spanish Castilian speakers (56 women, 51 men) were systematically recorded over two years at the Otorhinolaryngology Service of Hospital Universitario de Fuenlabrada, Spain. All participants had planned surgeries and were recorded in three time instants: 15 days before surgery (Ses. 1), 15 days post-surgery (Ses. 2), and 3 months post-surgery (Ses. 3). Inclusion criteria for controls encompass adults over 18 years of age scheduled for minor otorhinolaryngology surgeries, excluding those with previous vocal tract surgery, neck cancer, or disorders related to speech or voice. All participants underwent a clinical examination, including oral cavity and fiberoptic naso-endoscopic evaluations to confirm inclusion criteria. Patients who had pathological GRBAS (i.e., GRBAS > 0) or presented noticeable pathology in the vocal folds (i.e. lesions, scars, reflux) observed during nasofibroscopy were excluded from the study.

The total number of subjects is limited by the availability of patients received at the ENT service of Hospital de Fuenlabrada with the aforementioned pathologies. Moreover, the sample size has been determined to ensure significance in the previous studies^24,27,28^. The different sessions were defined to ensure a basal recording as close as possible to the surgical procedure, to evaluate the mid-term effect of the surgery, and to ensure that the patient was completely recovered from it.

The corpus comprises data from four distinct cohorts, categorised according to the type of surgery performed, all conducted by the same surgeon. Three out of the four cohorts underwent supraglottal tract surgeries, while the fourth had a minor surgery unrelated to the vocal tract:

- Tonsillectomy (*Tonsill*.): Involving the removal of inflamed or infected tonsils, usually performed in patients with recurrent tonsillitis who have not responded to other treatments. The average grade of tonsillitis for these participants was 3.1 on a scale of 0 to 4^30^. The dataset contains 25 patients belonging to this group.
- FESS (*FESS*.): A procedure to address nasal polyposis and chronic rhinosinusitis, which can cause nasal obstruction. This surgery involves the removal of inflamed nasal mucosal tissue and ethmoid bone cells to improve nasal airflow and reduce rinolalia. This cohort contains 27 patients.
- Septoplasty (*Sept*.): An intranasal procedure that corrects septal cartilage shape deformities, which can create airflow resistance during breathing and nasal ventilation. The dataset contains 29 patients in this group.
- Minor surgery (*Contr*.): This group underwent minor repair surgeries not related and not affecting the voice, speech, or vocal tract, thus serving as the control group. This cohort is built around 26 patients.

Demographic data for each patient were collected systematically during Ses. 1. These data encompassed several key details, including the patient’s age, which was represented as an integer, their self-declared gender indicated by a free string entry, and their height measured in centimetres.

Furthermore, the data included information on the patient’s diagnosis, documented as a free text blob field. Smoking habits were stored in a binary field, and the presence of OSA was marked similarly in a binary entry. For those who use CPAP therapy, its use was also documented. The professional use of voice (i.e., if voice is essential to their job) was saved in a binary field indicating “True/False”. The surgery date was also recorded as a datetime field (for the *Contr*. group, the date, when present, refers to a minor surgery not related to voice or speech). Additionally, doctor’s comments were stored in a free-text field written in Spanish. The *Tonsill*. cohort had specific information on tonsillar grade, while *FESS*. and *Sept*. had data related to Lund-Mackay scoring.

Regarding missing data, it should be noted that it was only observed in the last four columns: surgery date, doctor’s comments, tonsillar grade, and Lund-Mackay score. For more details on the extent of missing data, please refer to Table 1, which indicates the percentage of missing data in these columns for each cohort.

**Table 1:** Percentage of missing demographic data by cohort.

Cohort	Tonsillar Grade	Surgery date	Comment	Lund-Mackay score
*Tonsill*.	16.00 %	4.00 %	92.00 %	NA
*FESS*.	NA	0 %	96.30 %	7.41 %
*Sept*.	NA	3.44 %	93.10 %	96.55 %
*Contr*.	NA	7.69 %	NA	NA

#### Clinical Data

**Fig. 1:**
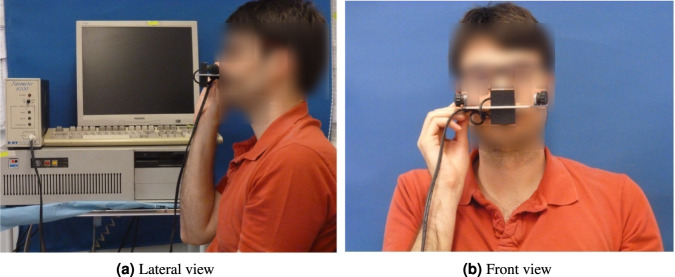
Use of the Nasometer. The device measures the nasality in percentage as a quotient of the nasal and oral energies.

At the beginning of each session, detailed clinical data were collected from each patient. This included annotating the patient’s weight. In addition, nasometry assessments were performed at the beginning of each session to measure nasality during sustained vowel /eh:/. For these measurements, the Nasometer II model 6450 was used, as seen in Fig. 1. Nasality was assessed as the ratio of acoustic energy that originates in the nasal tract compared to that of the oral tract. It was used as an indicator of the extent of the velopharyngeal opening during phonation. Lower values were associated with reduced nasality (hyponasalance), while higher values were indicative of increased nasality (hypernasalance). A higher nasalance score is expected after surgery (going from hyponasal to normal nasality).

Furthermore, a nasality questionnaire^9^, previously validated in the Spanish language (following a methodology similar to that of other adaptations^31,32^, was administered to gauge subjective perception of nasality. This questionnaire consisted of 13 items related to nasal symptoms, each of which was rated on a scale of 0 to 4. The questionnaire is available in the Appendix A. The cumulative scores from these items were used to derive a final total score included in the dataset.

**Fig. 2:**
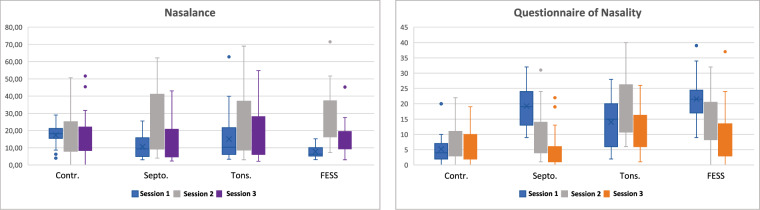
Objective nasalance (left) and subjective nasallity (right) obtained for the three sessions and for the four cohorts recorded: *Contr*., *Sept*., *Tonsill*., and *FESS*.

Table 2 presents the statistics for nasometry, the nasality questionnaire test, and weight for the different cohorts and sessions. Furthermore, Fig. 2 shows the box plots of the nasalance and nasality values obtained for each pathology and for the three recorded sessions. The box plots in Fig. 2 left correspond to the nasalance values obtained with the nasometer device; and those in Fig. 2 right correspond to the nasality values calculated from the self-assessment questionnaire presented to the patients.

**Table 2:** Weight, nasality questionnaire, and nasometry statistics in terms of mean ± std for the different cohorts and sessions.

Cohort	Weight (kg)	Nasality Questionnaire	Nasometry
	Ses 1		
	mean ± std	mean ± std	mean ± std
*Tonsill*.	77.12 ± 21.15	13.12 ± 8.07	15.04 ± 15.44
*FESS*.	83.30 ± 21.14	17.48 ± 9.95	13.53 ± 13.03
*Sept*.	79.90 ± 14.54	11.00 ± 6.77	11.29 ± 12.32
*Contr*.	77.00 ± 21.15	8.58 ± 6.20	16.27 ± 15.97
**Cohort**	**Ses 2**		
*Tonsill*.	68.40 ± 16.56	19.72 ± 9.74	19.33 ± 17.67
*FESS*.	83.15 ± 21.43	15.19 ± 9.06	20.79 ± 16.53
*Sept*.	79.17 ± 14.72	9.45 ± 7.20	16.73 ± 15.91
*Contr*.	73.08 ± 13.32	8.42 ± 5.32	14.61 ± 11.13
**Surgical Group**	**Ses 3**		
*Tonsill*.	70.04 ± 16.97	12.04 ± 6.25	18.71 ± 15.21
*FESS*.	83.96 ± 21.64	12.30 ± 8.99	16.60 ± 13.15
*Sept*.	78.79 ± 14.89	7.00 ± 7.17	14.07 ± 12.61
*Contr*.	72.39 ± 11.55	7.00 ± 5.40	15.37 ± 14.15

**Fig. 3:**
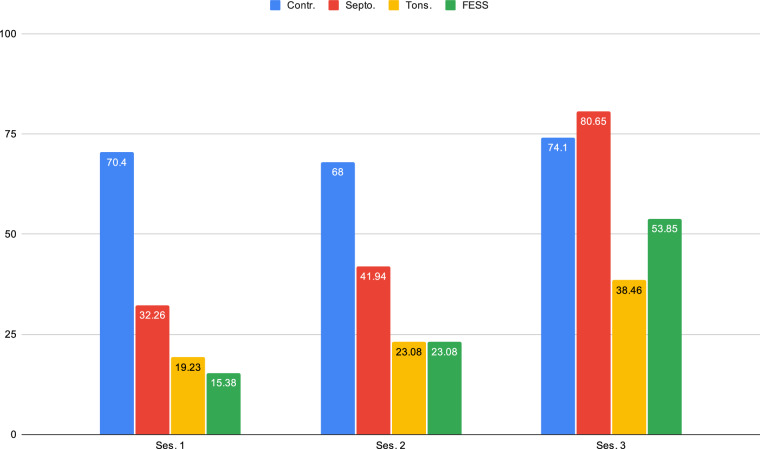
Evolution of the percentage of null GRBAS values per session. A clear increase after the surgery is shown.

Additionally, a GRBAS evaluation was performed during each visit as a subjective assessment of the quality of the patient’s voice. *Contr*. speakers who showed pathological GRBAS scores or had evident pathology in the vocal folds observed during nasofibroscopy were excluded from the study. Table 3 presents the statistics for subjective evaluations of GRBAS for the different groups and sessions. Furthermore, Fig. 3 shows the evolution of the null values obtained for the GRBAS score for each pathology and for the three recorded sessions.

**Table 3:** GRBAS values by cohort and session expressed in mean ± and standard deviation.

Surgical Group	Session 1				
	G	R	B	A	S
*Tonsill*.	1.00 ± 0.58	0.68 ± 0.48	0.00 ± 0.00	0.00 ± 0.00	0.32 ± 0.48
*FESS*.	1.07 ± 0.68	0.59 ± 0.57	0.07 ± 0.27	0.19 ± 0.40	0.22 ± 0.42
*Sept*.	0.69 ± 0.54	0.48 ± 0.51	0.00 ± 0.00	0.14 ± 0.35	0.03 ± 0.19
*Contr*.	0.00 ± 0.00	0.00 ± 0.00	0.00 ± 0.00	0.00 ± 0.00	0.00 ± 0.00
**Surgical Group**	**Session 2**				
*Tonsill*.	0.83 ± 0.64	0.67 ± 0.64	0.04 ± 0.20	0.00 ± 0.00	0.17 ± 0.38
*FESS*.	0.88 ± 0.53	0.60 ± 0.58	0.04 ± 0.20	0.08 ± 0.28	0.16 ± 0.37
*Sept*.	0.67 ± 0.55	0.33 ± 0.48	0.04 ± 0.19	0.11 ± 0.32	0.19 ± 0.40
*Contr*.	0.00 ± 0.00	0.00 ± 0.00	0.00 ± 0.00	0.00 ± 0.00	0.00 ± 0.00
**Surgical Group**	**Session 3**				
*Tonsill*.	0.72 ± 0.68	0.40 ± 0.50	0.08 ± 0.28	0.00 ± 0.00	0.24 ± 0.44
*FESS*.	0.58 ± 0.58	0.46 ± 0.51	0.04 ± 0.20	0.00 ± 0.00	0.08 ± 0.27
*Sept*.	0.29 ± 0.53	0.21 ± 0.42	0.00 ± 0.00	0.00 ± 0.00	0.04 ± 0.19
*Contr*.	0.00 ± 0.00	0.00 ± 0.00	0.00 ± 0.00	0.00 ± 0.00	0.00 ± 0.00

### Audio registration

The recording protocol, equipment, and environment were the same in all three sessions and across all groups. This meticulous approach was intended to produce high-quality data for subsequent analysis.

The audio recordings were conducted in a meticulously designed environment to ensure high-quality sound capture. Specifically, a soundproof, acoustically isolated, and carefully conditioned room was used, with a reverberation time consistently maintained below 0.25 seconds.

The audio equipment used for the recordings included a headset microphone, the AKG®C420, which operated at a sampling rate of 44,100 Hz. This microphone was connected to a 24-bit Soundblaster®Live sound card, which, in turn, was connected to a personal computer equipped with PRAAT®software^33^.

During the recording sessions, special attention was paid to ensure the comfort of the patients. They were asked to speak with a comfortable pitch and loudness to obtain the most natural and representative samples.

#### Script Creation

**Fig. 4:**
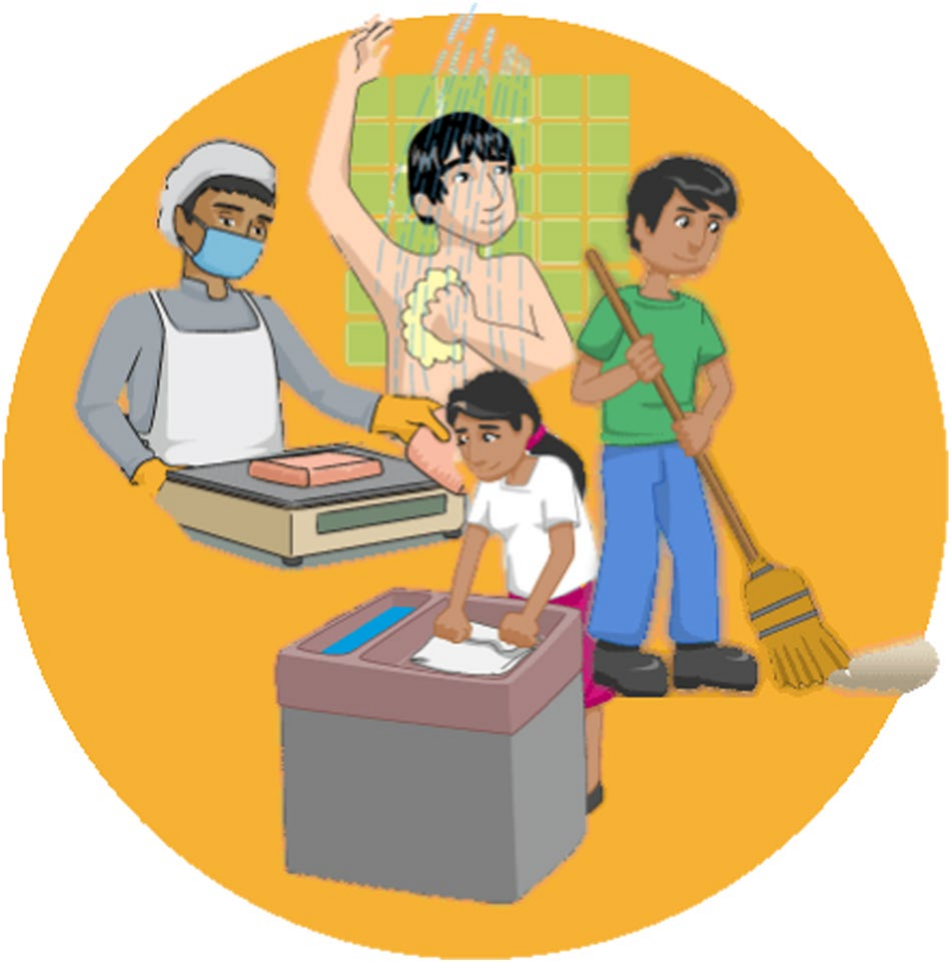
The free monologue is guided by asking the patient to describe the scenes presented in this illustration.

A specific protocol was designed to collect the voice and the speech of the speakers, ensuring a diverse range of vocal sounds and articulations, including sustained vowels, specific phrases, and spontaneous speech. The following acoustic material was recorded:

- Sustained vowel /a/: The patients were asked to phonate the sustained vowel /a / three times at a comfortable pitch and loudness, each with an approximate duration greater than 2 seconds.
- Sustained vowels /a/, /e/, /i/, /o/, and /u/: Patients were asked to phonate these five different sustained vowels, each with a duration of approximately 1 second, at a comfortable pitch and volume, and with short pauses to breathe between each vowel.
- TDU: Patients were instructed to recite four TDUs lasting approximately 10 seconds. These sentences are phonetically balanced and contain several nasalised sounds. The elocutions corresponding to the following sentences (in Spanish) were recorded:
  - *“Corre agua en el arroyo al crepúsculo”*, which in the International Phonetic Alphabet^34^ (IPA) corresponds to: [’ko ře ’a Ɣwa en el a ’řo yo al kre ’pus ku lo]
  - *“Calienta la casa el brasero y el hornillo de carbón”*, which in the IPA is: [ka ’ljen ta la ’ka sa el βra ’se ro i el or ’ni Lo ðe kar ’βon]
  - *“Es hábil un solo día”*, which in the IPA is: [es ’a βil un ’so lo ’ði a]
  - *“La mesa tiene ocho patas”*, which in the IPA is: [la ’mesa ’tjene ’ot∫o ’patas]
- Free monologue: As a final part of the recording process, patients were encouraged to describe a predetermined illustration depicting various activities of daily living, such as taking a shower or cleaning the house, as shown in Fig. 4. This free monologue was recorded for 1 minute. Specialists conducted manual transcriptions, making them accessible in both their raw and clean format, with the latter being carefully edited to exclude interjections, coughings, and background noises during silence periods.

#### Voice quality features

A set of voice quality features was extracted and included in the corpus. These features were extracted for the three repetitions of the 2-second sustained vowel /a /.

The open-source AVCA-ByO package^29^ was used to extract three subsets of characteristics, namely: i) amplitude and frequency perturbation features; ii) noise; and, iii) tremor. These voice quality features are stored in a .csv file. The parameters calculated are detailed in Table 4.

**Table 4:** Voice measurements extracted from the recordings of the /a / sustained vowels.

Parameter	Abbreviation	Unit Measure
**Pitch and Formants**		
Fundamental Frequency	F0	Hz
Formants	F1, F2, F3	Hz
Formant Bandwidths	F1_BW, F2_BW, F3_BW	Hz
Antiformants	antiF1, antiF2	Hz
Antiformant Bandwidths	antiF1_BW, antiF2_BW	Hz
**Perturbation Measures**		
Absolute Jitter	Jita	Seconds
Relative Jitter	Jitter	%
Relative Average Perturbation	Jitter RAP	%
Pitch Period Perturbation Quotient	Jitter PPQ	%
Smoothed Pitch Period Perturbation Quotient	Jitter sPPQ	%
Absolute Shimmer	SHDB	dB
Amplitude Perturbation Quotient	Shimmer APQ	dB
Smoothed Amplitude Perturbation Quotient	Shimmer sAPQ	dB
Cepstral Peak Prominence	CPP	dB
**Noise Parameters**		
Harmonics-to-Noise Ratio	HNR	dB
Cepstrum Harmonics-to-Noise Ratio	CHNR	dB
Glottal to Noise Excitation Ratio	GNE	Ratio
Normalised Noise Energy	NNE	dB
Noise-to-Harmonic Ratio	NHR	Ratio
Voice Turbulence Index	VTI	Percentage
Soft Phonation Index	SPI	Percentage
**Tremor Parameters**		
Fundamental Frequency Tremor Frequency	FFTR	Hz
Frequency Tremor Intensity Index	FTRI	Arbitrary Units
Amplitude Tremor Frequency	ATRF	Hz
Amplitude Tremor Intensity Index	ATRI	Arbitrary Units

Furthermore, the first three formants (F1, F2 and F3), the first two antiformants (antiF1 and antiF2), together with their respective bandwidths (BW), were extracted. The estimation of these characteristics was automatically carried out using the Kalman-based autoregressive moving average (KARMA) algorithm^35^. The interest in including the antiformant contours is based on their specific role in nasalisation. However, it should be noted that automatic tracking of antiformants still poses a significant challenge, given their typically weaker nature in comparison to their resonant counterparts during nasalised phonations. Thus, certain limitations are expected in the analysis of the antiformant contours provided. See the notes of the authors of the algorithm for more details about the limitations of the extracted antiformants^35^.

As a result, a .pkl file was generated for each audio file, containing the formant and antiformant contours; and their accompanying plot (in a .png file), where formants trajectories are shown, was included.

### Ethics declaration

The study was approved by the Ethics Review Board of the Hospital Universitario de Fuenlabrada (IRB: 18/11) in accordance with the Spanish Ethical Review Act. All participants completed the questionnaire and provided their written consent to participate in the study. Patients were individually identified with a code, which is different from the one used in the Hospital for their clinical histories, and no personal data was exchanged between the researchers who had access to the corpus. Patients were informed in detail of their rights and about the possibility of leaving the study at any time. All patients and controls were native Spanish speakers and followed the same experimental protocol. The otolaryngologist who performed the surgeries was the only person who got in contact with the patients, being also in charge of collecting the clinical data.

## Data Records

**Fig. 5:**
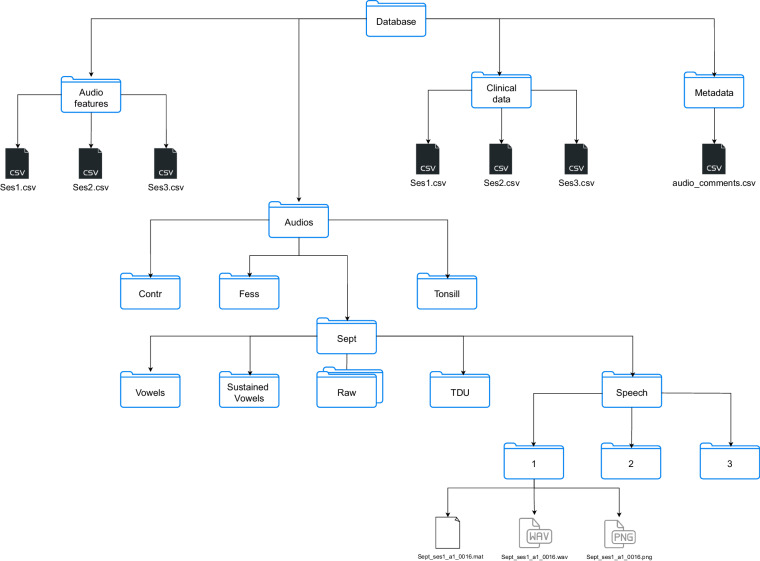
Database tree structure: hierarchical representation of the data records and their organization within the database. The structure displayed for the "Sept" folder is also applicable to the "Contr", "FESS", and "Tonsill" folders, but has been omitted for the sake of simplicity.

Accessing the data is facilitated through the Zenodo repository^36^. The structural representation of the data set can be seen in Fig. 5. The total size of the data set is 7.08 GB, comprising a collection of 3,800 audio files.

The data is distributed across four distinct folders, namely audio features, audios, metadata, and clinical data. In the subsequent sections, a detailed overview of the contents of each of these folders is provided.

### Voice Quality Features Repository

The "Audio features" folder contains all precomputed voice quality features. These features are available exclusively for the sustained vowel /a/. They are organised into three different .csv files named as follows: (i) the first session, which occurred 2 weeks before surgery, is labelled as "Ses1.csv"; (ii) the second session, which took place 2 weeks after surgery, is named "Ses2.csv"; and, (iii) the third session, which was 3 months after surgery, is indicated as "Ses3.csv". Each .csv file comprises 35 columns: the initial column indicates the patient ID, while the remaining 34 columns represent each of the precomputed voice quality features mentioned above.

### Audio Recordings Bank

The "Audios" folder contains all audio materials, including their formant and antiformant trajectories. This folder is further organised into four subfolders, each corresponding to a specific surgical procedure: "Contr", "FESS", "Sept", and "Tonsill". Within these subfolders, 5 subfolders are found.

Taking the "Sept" folder as an example, it contains 4 sub-subfolders, which are organised as follows:

- Folder "Vowels" contains sub-folders "A", "E", "I", "O", and "U". These subfolders contain one-second utterances of each vowel. Each of these folders is divided into subfolders labelled "1", "2", and "3" which correspond to the three recording sessions. Each audio file and their corresponding images and features follows the structure:
- "**SurgName_SessionNumber_AudioMaterial_IDPatient.Ext**" with **SurgName** being one out of: Contr, FESS, Sept, or Tonsill; the **SessionNumber** being one out of: Ses1, Ses2, or Ses3; the keyword **AudioMaterial** being one out of: a, e, i, o, u; the patient’s unique 4-digit ID in **IDPatient**; and **Ext** being one out of: wav, png or pkg.

**Fig. 6:**
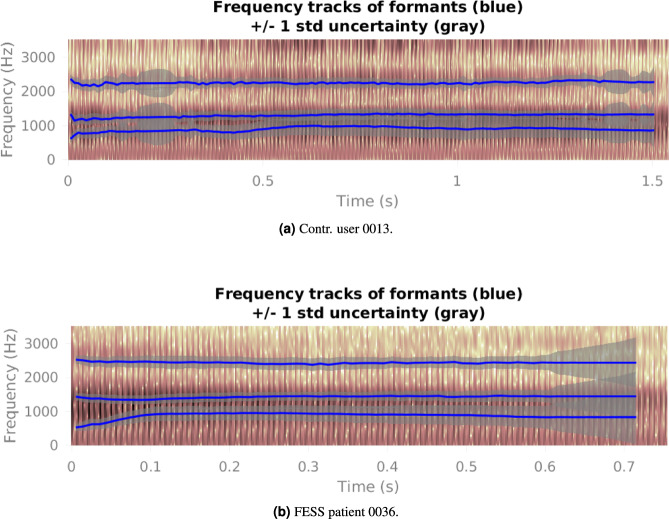
Formant and antiformant trajectories estimated with the KARMA algorithm for the sustained vowel /a/, first session, for a *Contr*. (**a**), and a *FESS* patient (**b**).

Within any of these sub-subfolders, the following files are found:

- "**SurgName_SessionNumber_AudioMaterial_IDPatient.wav**": This file contains the audio signal. For example, "Sept_ses1_a_0016.wav" represents the audio of the first session of the sustained vowel /a/ for patient 0016 who underwent septoplasty surgery.
- "**SurgName_SessionNumber_AudioMaterial_IDPatient.png**": The codification of the file is exactly the same as that of the .wav file but with .png as an extension. This image shows the formant trajectories. They are depicted on the corresponding wide-band spectrogram. Figure 6 is an example of these trajectories for patient 0063 of the *Tonsill*. group.
- "**SurgName_SessionNumber_AudioMaterial_IDPatient.pkl**": This file is structured as a Python dictionary. As detailed in Table 5, each .pkl file contains: (i) the normalised audio signal of length *L* × 1; (ii) the three first formants expressed by their mean, variance and bandwith; (iii) the two first antiformants expressed by their mean, variance, and bandwidth. Refer to usage_notes.ipynb as an example of how to read them.

**Table 5:** Dictionary contents for any **SurgName_SessionNumber_AudioMaterial_IDPatient.pkl** file where *L* is the length of the audio file and *W* the number of windows.

**Audio**		
Audio signal	*L* × 1	
**Formants**		
F1	Mean	*W* × 1
	Variance	*W* × 1
	Bandwidth	*W* × 1
	Mean	*W* × 1
F2	Variance	*W* × 1
	Bandwidth	*W* × 1
	Mean	*W* × 1
F3	Variance	*W* × 1
	Bandwidth	*W* × 1
**AntiFormants**		
AntiF1	Mean	*W* × 1
	Variance	*W* × 1
	Bandwidth	*W* × 1
	Mean	*W* × 1
AntiF2	Variance	*W* × 1
	Bandwidth	*W* × 1
**Params**		
peCoeff	0.7	
windowType	“Hamming”	
windowSizems	20	
windowOverlap	0.5	
lpcOrder	12	
zOrder	0	
fs	7000	
cepOrder	15	
cepType	1	
algFlag	2	

Regarding the “params” key they mean: peCoeff (Pre-emphasis coefficient), windowType (Window type - “Hamming”), windowSizems (Window length in milliseconds), windowOverlap (Window overlap fraction), lpcOrder (Number of AR coefficients), zOrder (Number of MA coefficients), fs (Downsampling frequency, in Hz), cepOrder (Number of cepstral coefficients), cepType (Type of cepstral coefficients - 1 for ARMA), and algFlag (Algorithm flag - 2 for extended Kalman smoother). Adjusting these parameters fine-tunes the algorithm for optimal analysis of the audio data.

- Folder "TDU" contains sub-folders "Agua", "Mesa", "Brasero", "Dia". These sub-subfolders contain the TDU corresponding to the aforementioned sentences, each lasting 10 seconds. The same organisational structure is maintained with .wav, .png, and .pkl files.
- Folder "Sustained vowels" contains subfolders "A1", "A2", "A3": These folders contain 2-second utterances of the sustained vowel /a/ with the same structure for the .wav, .png, and .pkl files. The voice quality features are available exclusively for this audio material.
- Folder "Raw": This folder contains two subfolders. The first subfolder, named "aeiou", includes the raw, unsplit versions of the one-second utterances of "A", "E", "I", "O", and "U". The second subfolder "ConcatenatedRead", contains the raw versions of the four TDU before they were split.

Audio recordings are saved in .wav files with a sampling frequency of 44.1 kHz. The wide-band spectrograms with the formant trajectories superimposed are saved as .png files, and the formant and antiformant sequences are stored in .pkl files, structured as dictionaries.

All audios were also manually edited to remove personal information (e.g., patients stating their names), as well as to eliminate word repetitions during the reading process, coughing, and background noises during silence periods.

### Clinical Data Repository

A set of clinical data was also recorded for each patient and session.

The "Clinical Data" folder contains the clinical data recorded following the protocol. It comprises three .csv files, one for each session, labelled as follows: (i) "Ses1.csv" for the first session; (ii) "Ses2.csv" for the second; and, (iii) "Ses3.csv" for the third session.

Each .csv file is structured around 28 columns: the initial column represents the patient’s ID; the second column specifies the surgical procedure; and the subsequent 12 columns include detailed clinical data as outlined in the methodology. The next 13 columns, one for each audio utterance, contain the file paths to all patient’s audio recordings for the corresponding session. Furthermore, each file contains demographic data for each patient. This .csv file is structured with the last 14 columns indicating the demographic data recorded according to the methodology outlined in the protocol.

### Metadata Bank

The "Metadata" folder contains a file with the doctor’s comments on each audio file.

The "Audio_comments.csv" file contains doctor’s comments (in Spanish) for each audio (when available), such as “the recording session has noise”, “the patient said ‘del’ instead of ‘de”’, or includes a flag that indicates if the audio required manual edition, for example, when the patient said their name. These comments are annotations and clarifications for each audio file. The .csv file consists of 27 columns, with the initial column indicating the patient’s ID, and the subsequent 26 columns containing comments for each audio material. For the five vowel sounds, namely /a/, /e/, /i/, /o/, and /u/, the comments are combined into a single column named "aeiou".

## Technical Validation

The authors extensively explored different subsets of the corpus, which have been the basis for several publications^24,27,28^.

The technical validation of the described data set was carried out by analysing variations in clinical data between the control and surgery groups, before and after surgical procedures. Statistical tests, including t-Student for normally distributed quantitative features, the Wilcoxon test for non-normality distributed quantitative features, and the Fisher exact test for categorical variables, were employed. Table 6 highlights statistically significant variations in subjective measurements (GRBAS and questionnaire), and objective nasalance measurements, highlighting the differences between the control and pathological groups.

**Table 6:** Statistical comparison of each surgery group against the *Contr*. group through p-values before the surgical procedure, i.e., Ses. 1.

VARIABLE	*Contr*.	*Sept*.	p-Value	*Tonsill*.	p-Value	*FESS*.	p-Value
**SEX**			**<0.001**		>0.05		>0.05
Male (%)	10 (37.04)	25 (80.65)		5 (19.23)		12 (46.15)	
Female (%)	17 (62.96)	6 (19.35)		21 (80.77)		14 (53.85)	
**AGE** (**mean** ± **std**)	49.44 ± 12.27	35.32 ± 13.68	**<0.001**	28.77 ± 10.63	**<0.001**	49.35 ± 11.26	>0.05
**SMOKER**			>0.05		>0.05		>0.05
Yes (%)	11 (40.74)	8 (25.81)		9 (34.62)		9 (34.62)	
No (%)	16 (59.26)	23 (74.19)		17 (65.38)		17 (65.38)	
**PROFESSIONAL VOICE**			>0.05		>0.05		>0.05
Yes (%)	8 (29.63)	6 (19.35)		6 (23.08)		7 (26.92)	
No (%)	19 (70.37)	25 (80.65)		20 (76.92)		19 (73.08)	
**GRBAS** (**mean** ± **std**)	0.63 ± 1.08	1.32 ± 0.97	**<0.05**	1.92 ± 1.20	**<0.001**	2.15 ± 1.25	**<0.001**
**N. QUESTIONNAIRE** (**mean** ± **std**)	5.15 ± 4.19	19.26 ± 6.63	**<0.0**5	12.92 ± 7.97	**<0.05**	21.54 ± 7.35	**<0.001**
**NASALANCE** (**mean** ± **std**)	17.13 ± 6.24	10.61 ± 6.25	**<0.05**	15.08 ± 13.36	**<0.05**	7.77 ± 3.59	**<0.05**

The numbers indicate the total number of occurrences, with percentages in parentheses.

Consequently, a detailed examination of the variations between sessions in objective nasalance, subjective self-assessment nasality questionnaires, and GRBAS values were conducted. This analysis revealed notable differences between sessions, as described in Table 7. In the *Contr*. group, no significant changes were observed in objective or subjective measurements. However, a statistically significant variation was identified in the subjective measurement of nasality between the first and last sessions for both the *FESS*. and *Sept*. groups, with p-values < 0.05 and < 0.001, respectively (Fig. 2).

**Table 7:** Intra-group variation of GRBAS, nasality questionnaire and nasalance measurements by session.

Cohort	Variable	Ses. 1	Ses. 2		Ses 3	
		Value	Value	p-Value	Value	p-Value
***Tonsill***.	GRBAS 0 (%)	5 (19.23)	6 (23.08)	**<0.05**	10 (38.46)	**<0.05**
	1 (%)	0 (0.0)	0 (0.00)		0 (0.0)	
	2 (%)	17 (65.38)	15 (57.69)		15 (57.69)	
	3 (%)	0 (0.0)	1 (3.85)		0 (0.0)	
	4 (%)	4 (15.38)	4 (15.38)		1 (3.85)	
	Nasality Questionnaire	12.92 (7.97)	24.27 (9.82)	**<0.01**	11.88 (6.18)	>0.05
	Nasalance Nasometer	15.08 (13.36)	23.89 (17.60)	**<0.01**	18.24 (15.09)	>0.05
***FESS***.	GRBAS 0 (%)	4 (15.38)	6 (23.08)	**<0.05**	14 (53.85)	**<0.05**
	1 (%)	0 (0.0)	0 (0.0)		0 (0.0)	
	2 (%)	16 (61.54)	18 (69.23)		12 (46.15)	
	3 (%)	0 (0.0)	0 (0.0)		0 (0.0)	
	4 (%)	6 (23.08)	2 (7.69)		0 (0.0)	
	Nasality Questionnaire	21.54 (7.35)	14.92 (9.14)	>0.05	9.81 (6.39)	**<0.01**
	Nasalance Nasometer	7.77 (3.59)	27.92 (14.67)	**<0.001**	16.08 (10.79)	>0.05
***Sept***.	GRBAS 0 (%)	10 (32.26)	13 (41.94)	**<0.05**	25 (80.65)	**<0.05**
	1 (%)	2 (6.45)	1 (3.23)		0 (0.0)	
	2 (%)	18 (58.06)	16 (51.61)		6 (19.35)	
	3 (%)	1 (3.23)	0 (0.00)		0 (0.0)	
	4 (%)	0 (0.0)	1 (3.23)		0 (0.0)	
	Nasality Questionnaire	19.26 (6.63)	9.71 (7.05)	>0.05	4.52 (5.39)	**<0.001**
	Nasalance Nasometer	10.61 (6.25)	26.36 (19.26)	**<0.001**	13.36 (12.23)	>0.05
Contr.	GRBAS 0 (%)	19 (70.4)	17 (68)	>0.05	20 (74.1)	>0.05
	1 (%)	1 (3.7)	1 (4)		0 (0.0)	
	2 (%)	6 (22.2)	6 (24)		6 (22.2)	
	3 (%)	0 (0.0)	0 (0.0)		0 (0.0)	
	4 (%)	1 (3.7)	1 (A)		1 (3.7)	
	Nasality Questionnaire	5.15 (4.19)	7.70 (5.17)	>0.05	6.74 (4.96)	>0.05
	Nasalance Nasometer	17.13 (6.24)	16.52 (11.70)	>0.05	16.96 (13.31)	>0.05

The p-value is calculated using the T-student test for quantitative variables and the Fisher exact test for the categorical ones. The numbers indicate the total number of occurrences, with percentages in parentheses.

Significant variations in GRBAS measurements are evident in all surgical groups, each with p-values < 0.05. This underscores the discernible differences between the control and surgery voices. A notable trend, as illustrated in Fig. 3, is the consistent increase of null values in GRBAS measurements for all surgical procedures, indicating that their voice is improving, compared to the constant trend observed in the *Contr*. group.

## Usage Notes

The Python data management scripts are available in the GitHub®repository cited in the Code Availability section.

For practical guidance and hands-on demonstration, the corpus contains a Jupyter®notebook named "Usage_notes.ipynb". This notebook includes a comprehensive code section that illustrates the process of reading audio files and normalising the data. Additionally, it features a simple yet illustrative experiment. In this experiment, Mel-Frequency Cepstral Coefficients are computed for the /a/ sustained vowels corresponding to the first session, and a simple Random Forest classifier is trained to distinguish between the control and pathological groups. This simple experiment achieves 74% of accuracy, and provides a practical illustration of how to work with the dataset, serving as a starting point for further exploration and analysis. There is no aim to maximise the classification accuracy with this simple experiment.

## Data Availability

All codes used for data set preprocessing and cleaning can be accessed in a specific GitHub repository (https://github.com/BYO-UPM/CUCO_Database). Additionally, the aforementioned Jupyter notebook is included as a reference in the same repository. The KARMA algorithm is available as an open-source package in a specific GitHub repository (https://github.com/BYO-UPM/Formant-Tracking). The AVCA-ByO toolbox is also available as an open-source package^29^.

## A Nasal Questionnaire

This section presents the subjective self-assessment nasality questionnaire used. Each item has five possible answers and each is scored with a value from 1 to 5. The total grade assigned to each patient is calculated as the sum of the partial scores for each item evaluated.

1) Do you think that when you speak, it sounds as if you had a stuffy nose?

Never Rarely Sometimes Often Always

2) Do you think that when you speak, it sounds like a lot of sound coming from your nose?

Never Rarely Sometimes Often Always

3) Does your voice get tired during the day?

Never Rarely Sometimes Often Always

4) Do you have the sensation of having a dry throat during the day?

Never Rarely Sometimes Often Always

5) Do you have a sensation of tickling in the throat?

Never Rarely Sometimes Often Always

6) Do you have phlegm?

Never Rarely Sometimes Often Always

7) Does it make you uncomfortable to speak for an extended period?

Never Rarely Sometimes Often Always

8) Do people have difficulty understanding you because of your voice?

Never Rarely Sometimes Often Always

9) Does your voice affect you at work?

Never Rarely Sometimes Often Always

10) Does your voice affect you in social activities?

Never Rarely Sometimes Often Always

11) Do your family, friends, or coworkers comment that they are bothered by your voice?

Never Rarely Sometimes Often Always

12) Are you concerned about your voice?

Never Rarely Sometimes Often Always

13) Are you unhappy with your voice?

Never Rarely Sometimes Often Always

**(To be completed after surgery)**

14) How different do you think your voice is after surgery?

- Not different at all
- Slightly different
- Moderately different
- Considerably different
- Severely different

15) If it is different, do you think your voice is:

- Slightly better
- Better
- Slightly worse
- Worse
- Neither better nor worse
- Not sure
